# Comparison of Clinical Scoring Systems in the Management of Patients with Microangiopathic Hemolytic Anemia and Thrombocytopenia

**DOI:** 10.4274/tjh.galenos.2020.2020.0348

**Published:** 2021-02-25

**Authors:** Mehmet Baysal, Elif Ümit, Hakkı Onur Kırkızlar, Ahmet Muzaffer Demir

**Affiliations:** 1 Trakya University Faculty of Medicine, Department of Hematology, Edirne, Turkey

**Keywords:** Thrombotic thrombocytopenic purpura, PLASMIC score, Thrombotic microangiopathy, French score, Bentley score

## Abstract

**Objective:**

Several clinical scoring systems have been developed for the differential diagnosis of thrombotic microangiopathies (TMAs), all to predict and identify patients with ADAMTS13 deficiency and to start treatment as soon as possible. The first scoring system in this regard was the Bentley score, and the French score and PLASMIC score were developed afterwards.

**Materials and Methods:**

We aimed to evaluate the laboratory parameters and clinical features of patients who underwent plasma exchange with a prediagnosis of TTP at our clinic between 2007 and 2019 and whose ADAMTS13 enzyme levels were measured and to compare the findings with the scoring systems.

**Results:**

Data of 35 patients were evaluated. Twelve patients were evaluated as high risk according to all three scoring systems. A statistically significant relation was observed between all three scoring systems and ADAMTS13 levels.

**Conclusion:**

A moderate correlation was found between all three scoring systems and ADAMTS13 levels. We observed similar potential strength of all three scoring systems to predict TTP among other TMAs and we conclude that they are applicable in daily practice.

## Abstract (tr)

**Amaç:**

Trombotik mikroanjiyopatilerin (TMA) ayırıcı tanısı yapabilmek ve ADAMTS13 eksikliği olan hastaları ayırt edebilmek için çeşitli klinik skorlama sistemleri geliştirilmiştir. Bu konuda ilk geliştirilen skorlama sistemi Bentley skoru olup; French ve PLASMIC skorları daha sonra geliştirimiştir.

**Gereç ve Yöntemler:**

2007-2019 yılları arasında kliniğimizde trombotik trombositopenik purpura (TTP) ön tanısıyla plazma değişimi yapılan ve ADAMTS13 enzim düzeyi ölçülen hastaların klinik özellikleri ve laboratuvar verilerini her üç skorlama sistemine göre karşılaştırılmayı amaçladık.

**Bulgular:**

Otuz beş hastanın verileri geriye dönük olarak değerlendirildi. Her üç skorlama sistemine göre 12 hasta yüksek riskli olarak değerlendirildi. Skorlama sistemleri ve ADAMTS13 seviyeleri arasında istatistiksel olarak anlamlı bir ilişki gözlendi. Ayrıca, skorlama sistemleri ile ADAMTS13 seviyeleri arasında orta düzeyde bir korelasyon bulundu.

**Sonuç:**

Her üç skorlama sisteminin günlük pratikte kolayca uygulanabilir olması ile birlikte; TTP ön tanısı olan hastaları ayırt etmede benzer bir potansiyel güçte olduğunu gözlemledik.

## Introduction

Thrombotic thrombocytopenic purpura (TTP) is a thrombotic microangiopathy (TMA) characterized by microangiopathic hemolytic anemia and thrombocytopenia developed due to deficiency of ADAMTS13 (a disintegrin and metalloprotease with thrombospondin type 1 repeats, member 13) [1]. The ADAMTS13 enzyme is responsible for controlling the length of the von Willebrand factor (vWF) multimers and the absence of ADAMTS13 activity causes the formation of very large (ultra-large) vWF multimers, which have an increased tendency to attach to the endothelium [2]. The difference between TTP and other TMAs including hemolytic uremic syndrome (HUS), atypical HUS, complement-mediated TMA, and drug-induced TMA is basically the deficiency of ADAMTS13 enzyme activity [2,3]. Accepted as a rare disease, the estimated and reported incidence of TTP is 2-3 persons per 1 million with a female predominance [4]. The treatment of TTP entails emergency plasma exchange and, to overcome that challenging emergency situation, several clinical scoring systems of probability have been developed and proposed [5]. With the use of these clinical scoring systems it is aimed to identify patients with ADAMTS13 deficiency and to start plasma replacement treatment as soon as possible.

The first scoring system in this regard was the Bentley score, which was followed by the French score and PLASMIC score. The Bentley score was developed with a retrospective analysis of 110 patients and 5 laboratory parameters were suggested to be significantly correlated with ADAMTS13 levels [6]. Validation of the Bentley score was performed afterwards with a cohort with 84 patients [7]. The second score was called the French score, based on the analysis of 214 patients and including platelet count, creatinine level, and antinuclear antibody (ANA) [8]. Although these scoring systems were based on studies with significant numbers of patients, they were never popular in daily practice. The last one was called the PLASMIC score, developed based on the data of the Harvard TMA registry and validated with several studies [5,9,10,11].

Determination of the ADAMTS13 level is essential for the definitive diagnosis of TTP as well as for monitoring the course of the disease. Since the determination of ADAMTS13 enzyme activity is performed in specialized centers and laboratories, requiring a significant amount of time, it has become a goal for clinicians to predict possible TTP patients in order to start the lifesaving treatment.

In our study, we aimed to evaluate the laboratory parameters and clinical features of patients who underwent plasma exchange with a prediagnosis of TTP at our clinic between 2007 and 2019 and whose ADAMTS13 enzyme levels were measured and to compare those findings with the scoring systems.

## Materials and Methods

Our study was designed as a retrospective study. Data of patients who underwent plasma exchange with a prediagnosis of TTP between 2007 and 2019 were evaluated. The Bentley score, French score, and PLASMIC score were calculated based on the data of patient files. Calculations and interpretations of the clinical scoring systems are summarized in Table 1.

Samples for ADAMTS13 activity level determination were collected before plasma exchange initiation from a citrated plasma sample. The samples were centrifuged and stored at -80 °C before assessment. The enzyme-linked immunosorbent assay (ELISA) method was used to measure ADAMTS13 activity. Severe ADAMTS13 deficiency was defined as an ADAMTS activity level lower than 10% [1,12,13,14]. In statistical analysis, continuous variables were represented as mean ± standard derivation. For categorical variables, the chi-square test was used. Correlation analysis was performed using Spearman tests.

### Statistical Analysis

SPSS 22.0 (IBM Corp., Armonk, NY, USA) was used for statistical analysis and a two-sided p-value less than or equal to 0.05 was considered as statistically significant.

Our study was carried out in accordance with the 1964 Declaration of Helsinki and its later amendments. Ethics committee approval was obtained from the local ethics board (TUTF-BAEK 2020-214).

## Results

A total of 35 patients were evaluated; 25 patients were female and 10 patients were male. The ages of the patients ranged from 20 to 84 years with an average age of 45.91. Demographic and laboratory features of the patients are given in Table 2. According to the PLASMIC scores, 13 patients were evaluated as high risk, 10 patients as moderate risk, and 12 as low risk. When the Bentley and French scoring systems were evaluated, by both scoring systems 19 patients were evaluated as high risk, 11 patients as moderate risk, and 5 patients as low risk (Table 3). A total of 12 patients were evaluated as high risk according to all 3 scoring systems. In addition, patients were divided into 2 groups according to ADAMTS13 activity levels of <10% and >10%, and clinical scoring systems were compared for these groups. In statistical analysis using the chi-square method, a significant relationship was found between all 3 scoring systems and ADAMTS13 levels (p<0.05) (Table 4). In addition, the relationship between scoring systems and ADAMTS13 levels was evaluated by Spearman correlation analysis and a strong positive correlation was found between all 3 scoring systems and ADAMTS13 levels (Table 5). In the final evaluation of the patients, 23 were diagnosed with TTP (for ADAMTS activity levels of <10%), 4 were diagnosed with HUS, 3 were diagnosed with primary autoimmune/rheumatologic disease, 2 were diagnosed with sepsis/infection, 2 were diagnosed with atypical HUS, and 1 was diagnosed with drug-induced thrombotic microangiopathy.

## Discussion

TTP was first described by Moschcowitz in 1925. He reported a 16-year-old girl presenting with bleeding, hematuria, and neurologic symptoms [15]. Later, in a landmark paper in 1966, it was defined as a constellation of symptoms including microangiopathic anemia, thrombocytopenia, fever, neurologic signs, and renal failure. Fragmented erythrocytes also called schistocytes were observed on peripheral blood smears as an indicator of microangiopathic hemolytic anemia [16]. The pathophysiology was unclear until the 1990s, when the contribution of vWF due to deficiency of ADAMTS13 enzyme, which cleaves the ultra-large multimers, was discovered to be the missing piece [16].

In spite of these developments in the pathophysiology and pathobiology of TMAs, the clinical picture of TTP remains challenging for physicians. Few of the five symptoms of the classical pentad were clinically demonstrated in patients [3,12,17]. Although ADAMTS13 enzyme level measurement remains the most essential parameter, assessment of ADAMTS13 level is only possible in specialized laboratories, requiring time to obtain the sample and perform the analysis itself. This remains the most challenging issue around the world. For this reason, various clinical predictive scoring systems have been developed to diagnose TTP. The main aim here is to distinguish patients who will benefit from emergency plasma exchange treatment until the laboratory assessment of ADAMTS13. Despite the fact that plasma exchange is the most successful treatment for TTP to date, central catheter insertion is required and plasma exchange therapy itself may have serious side effects. Therefore, evaluation of clinical scoring systems in TTP-suspected patients remains critically important.

The PLASMIC score was designed and externally validated in a cohort of 214 patients. In that analysis, the authors stated that both the Bentley score and French score, though much easier to calculate, have weaknesses in detecting TTP, including their lack of external validation [5]. The autoimmune nature of the disease and the promising results obtained by adding rituximab shows us that ANA positivity, which was included in the French score, could be a reasonable marker [18,19,20]. In a recent analysis with limited patients, it was also suggested that adding the ratio of lactate dehydrogenase level/upper normal limit could strengthen the value of the PLASMIC score [21]. Considering all this together, while no scoring system is definitive, each has its own strengths and weaknesses. Therefore, physicians and clinicians should determine how to interpret them and use them in certain situations.

The small sample size is a weakness of our study. However, for rare diseases such as TTP, it is hard to perform studies with larger sample sizes. TTP diagnosis and treatment are urgent emergencies and action must be taken immediately. With these scoring systems, rapid plasmapheresis treatment is recommended for high-risk patients. In our study with a limited number of patients, we found that there was no difference between the scoring systems. Our findings need to be evaluated with studies involving more patients.

## Figures and Tables

**Table 1:**
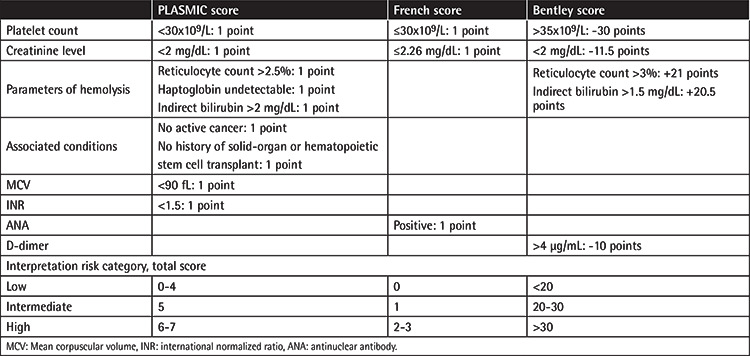
Interpretation of clinical scoring systems for ADAMTS13 deficiency.

**Table 2:**
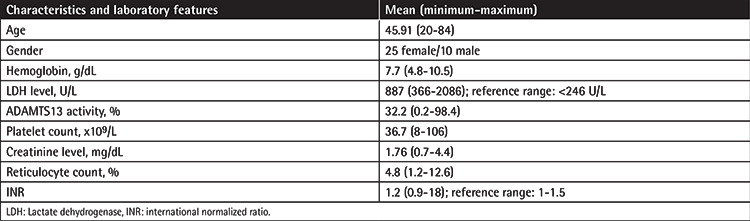
Demographic and laboratory features of the study population.

**Table 3:**
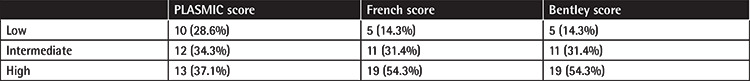
Distribution of patients by clinical scoring systems.

**Table 4:**
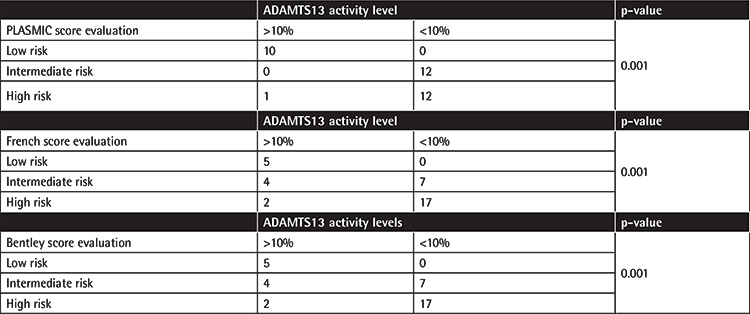
Distribution of PLASMIC score, French score, and Bentley score categories according to ADAMTS13 levels.

**Table 5:**
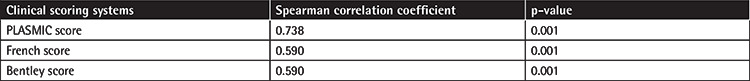
Spearman correlation analysis results between scoring systems and ADAMTS13 levels.
